# Supplementary figures and images for: Targeted recruitment of USP15 enhances CTLA4 surface levels and restricts its degradation
Source: Life Sci Alliance. 2026 Jan 22;9(4):e202503563. doi: 10.26508/lsa.202503563 (PMC12827578; doi:10.26508/lsa.202503563)

**Figure 2 A**

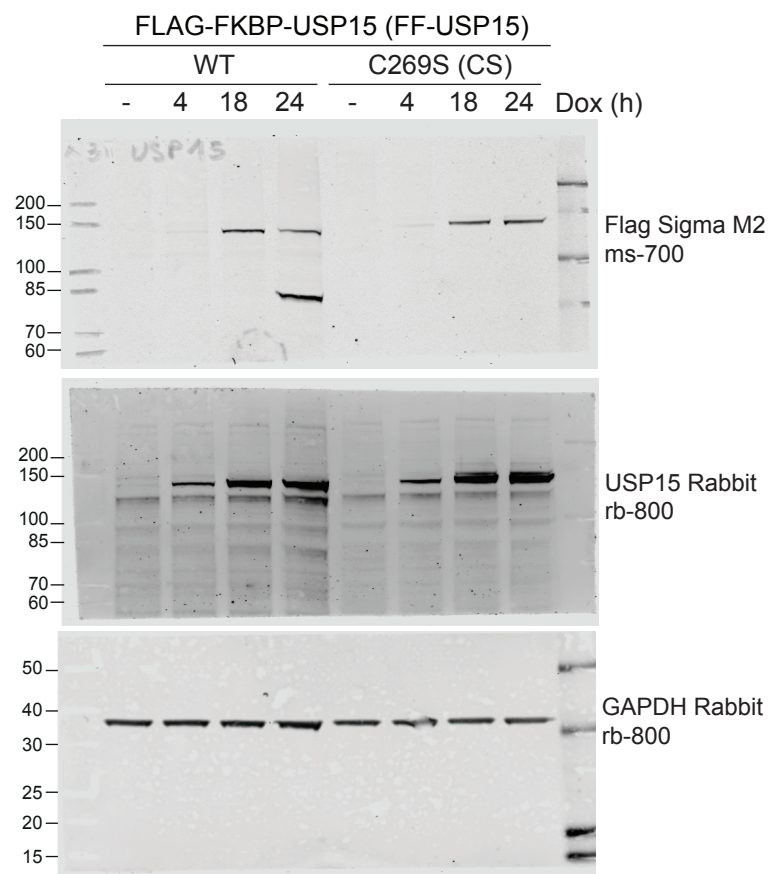

Figure 2 D

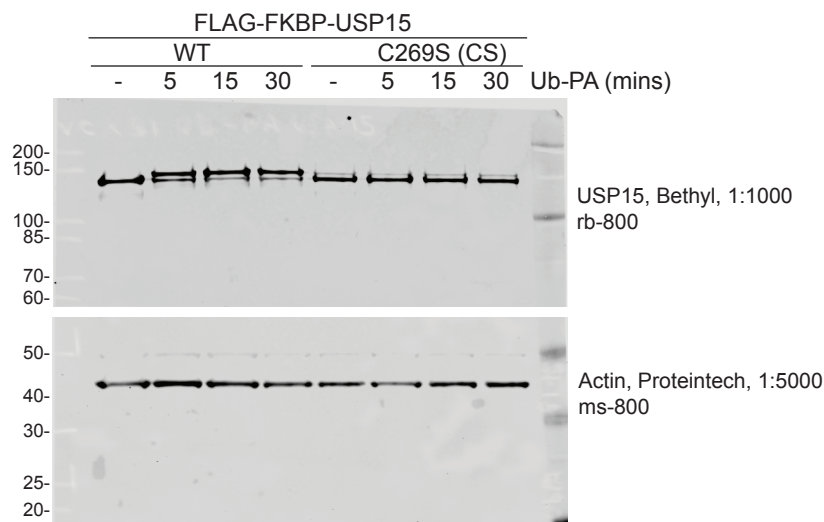

Ponceau

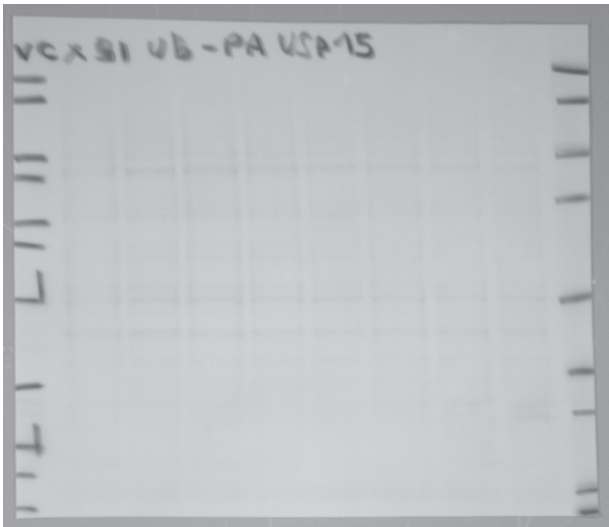

Supplement: Supplementary file 1 [file LSA-2025-03563_SdataF2.pdf]

Figure S1A

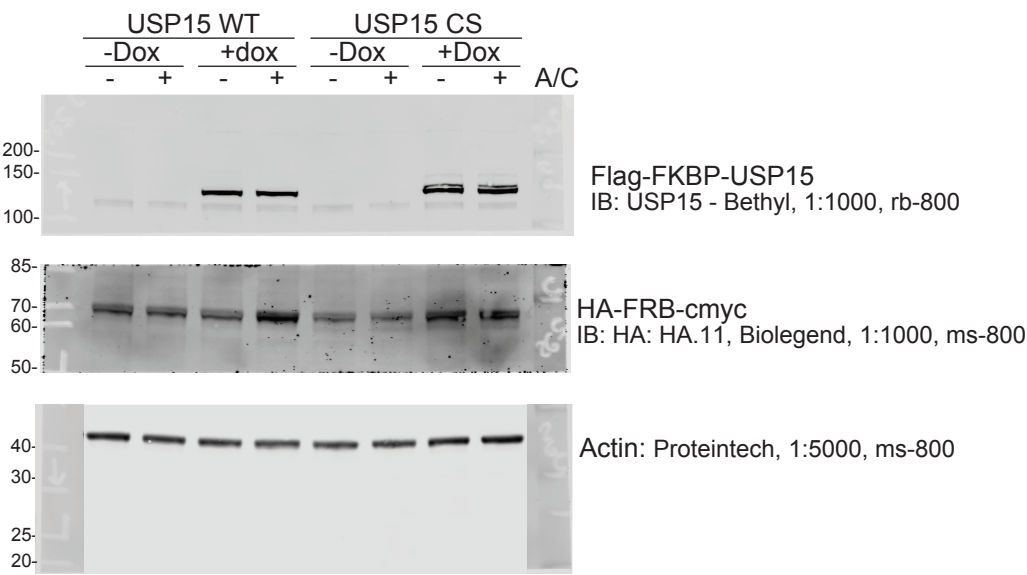

Supplement: Supplementary file 2 [file LSA-2025-03563_SdataFS1.pdf]

Figure 3 B

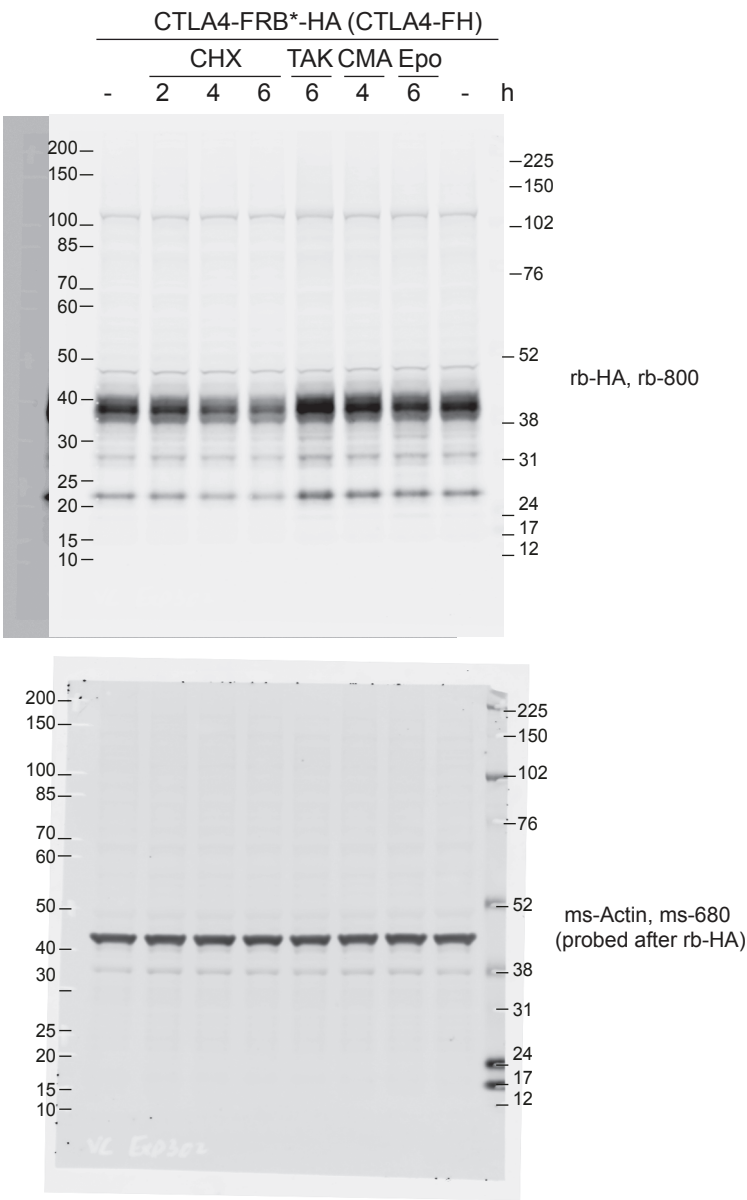

Figure 3 C

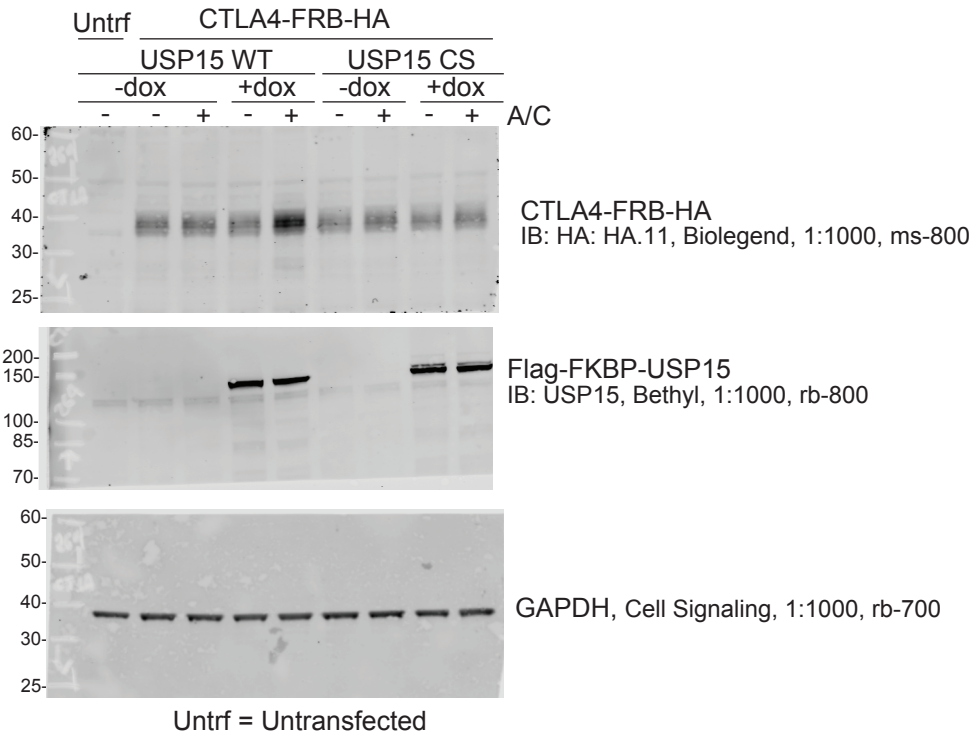

Figure 3 E, F

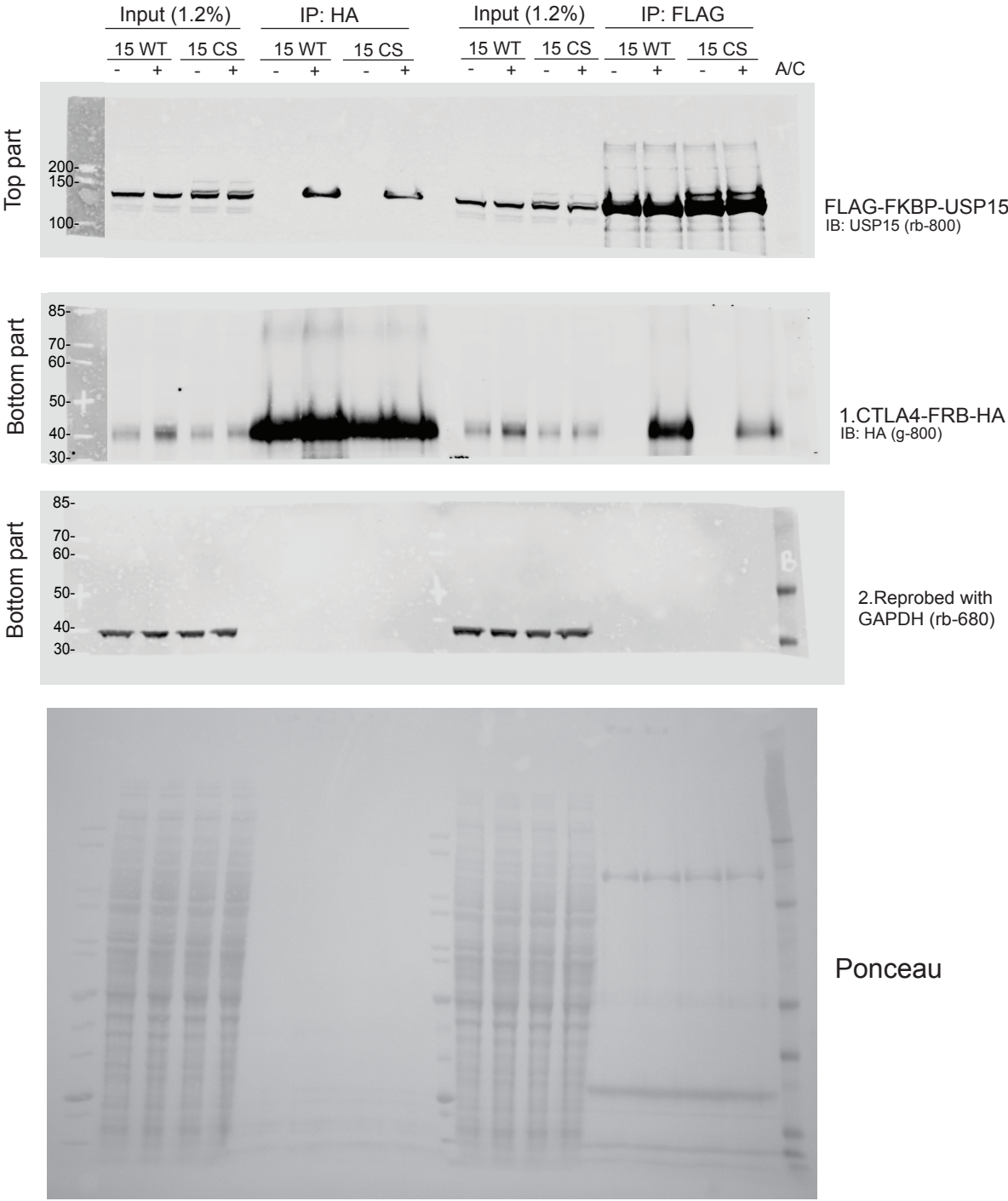

Supplement: Supplementary file 3 [file LSA-2025-03563_SdataF3.pdf]

**Figure S2A**

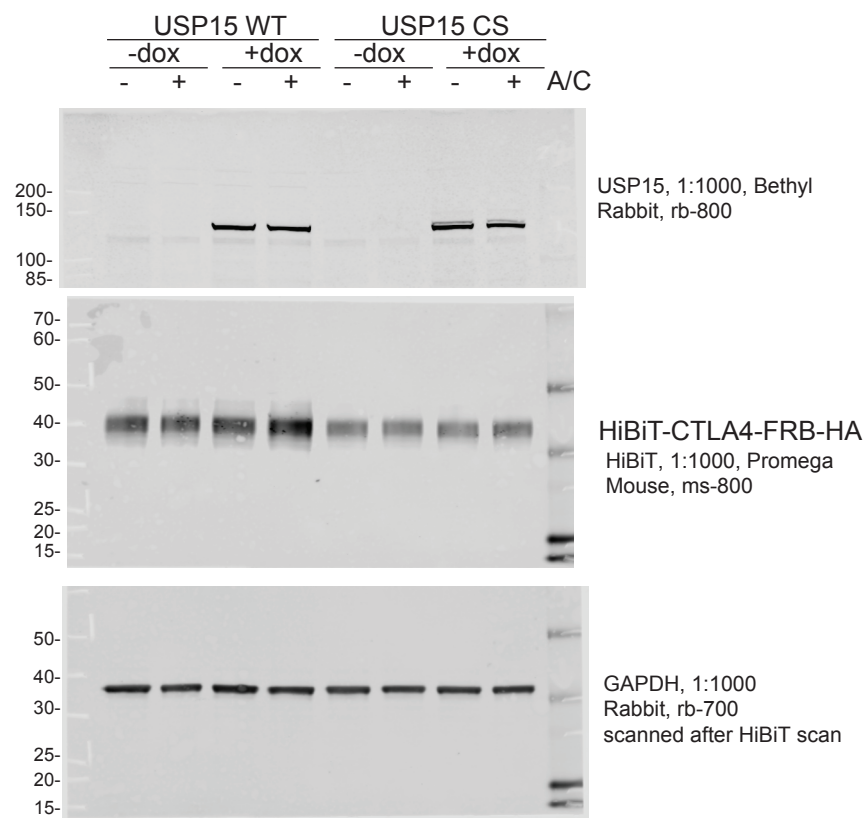

Figure S2B

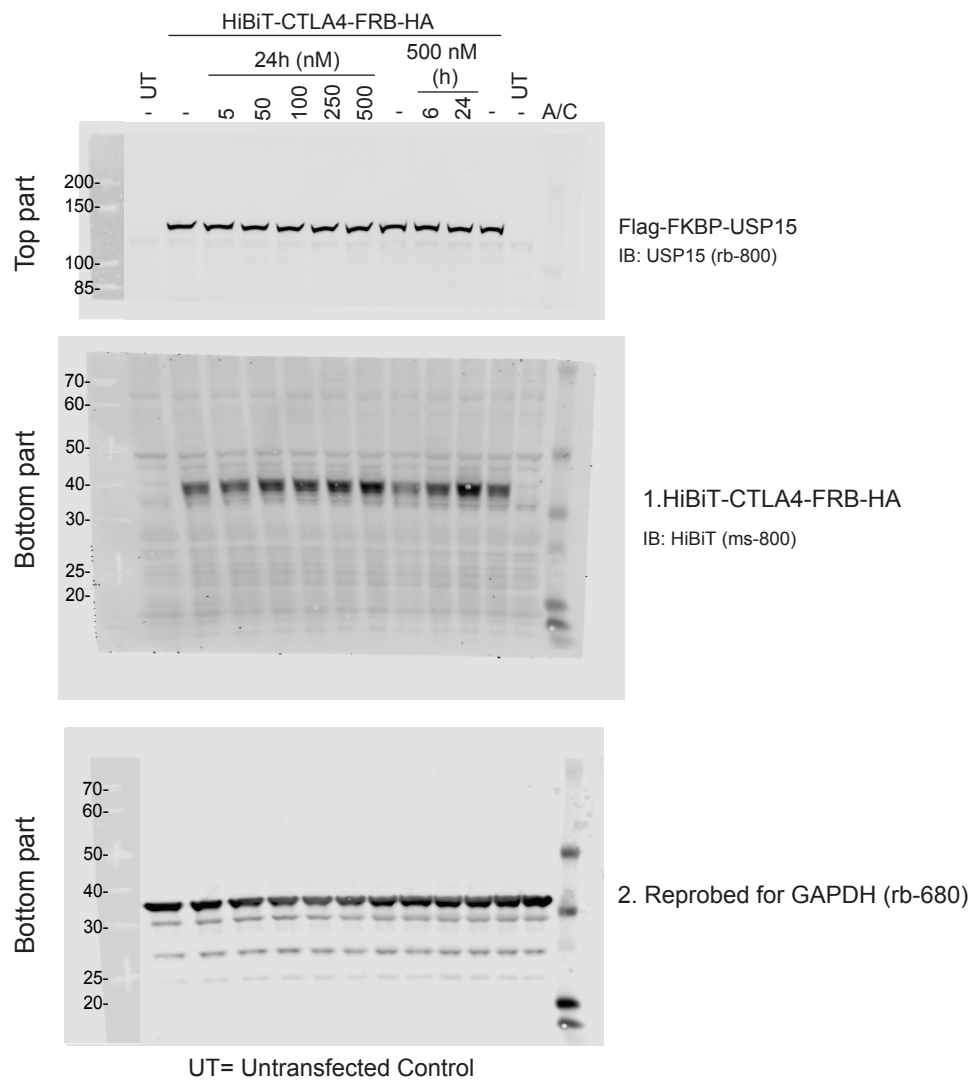

Supplement: Supplementary file 4 [file LSA-2025-03563_SdataFS2.pdf]

Figure 5B

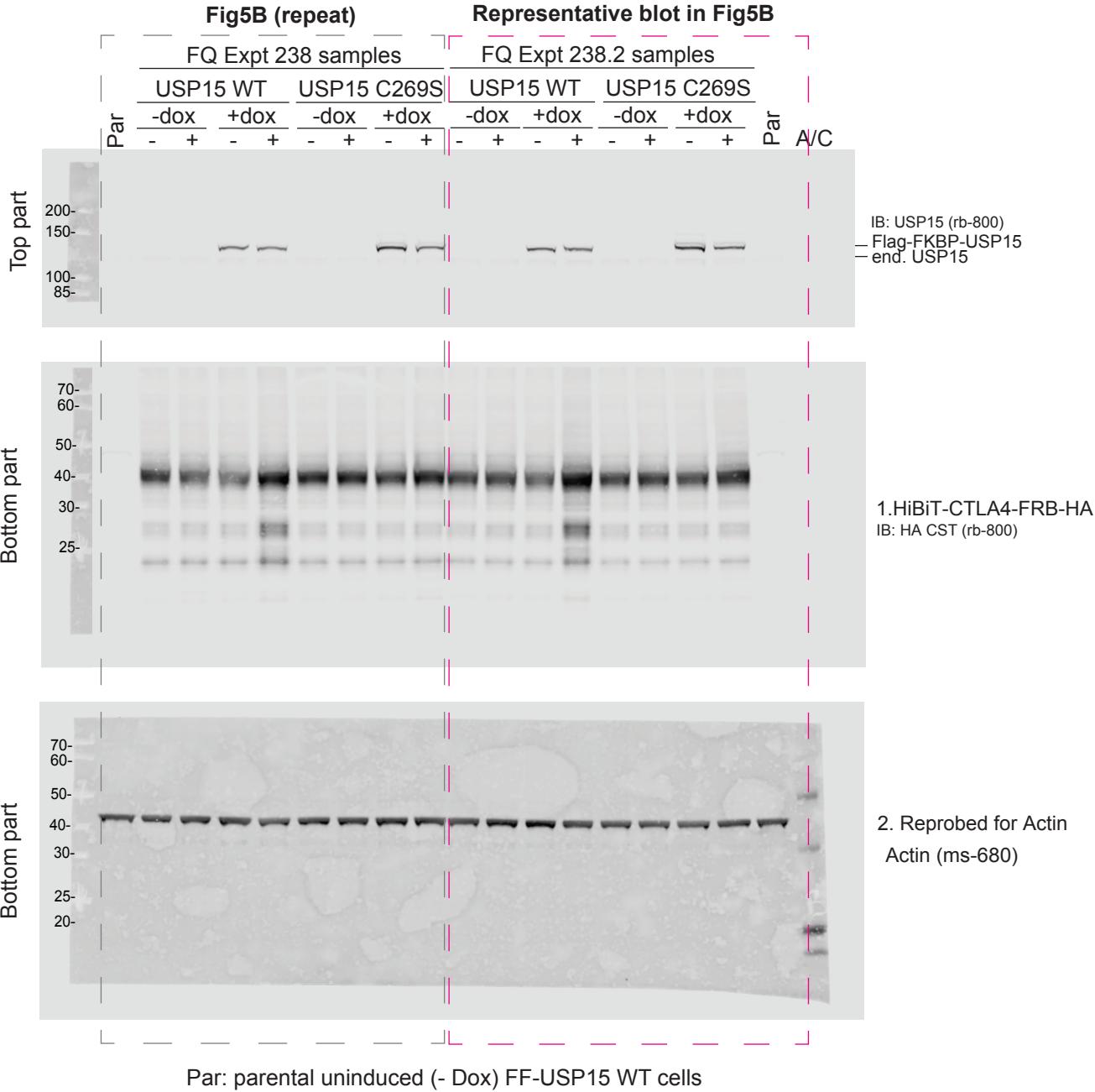

Supplement: Supplementary file 5 [file LSA-2025-03563_SdataF5.pdf]

Figure S3A

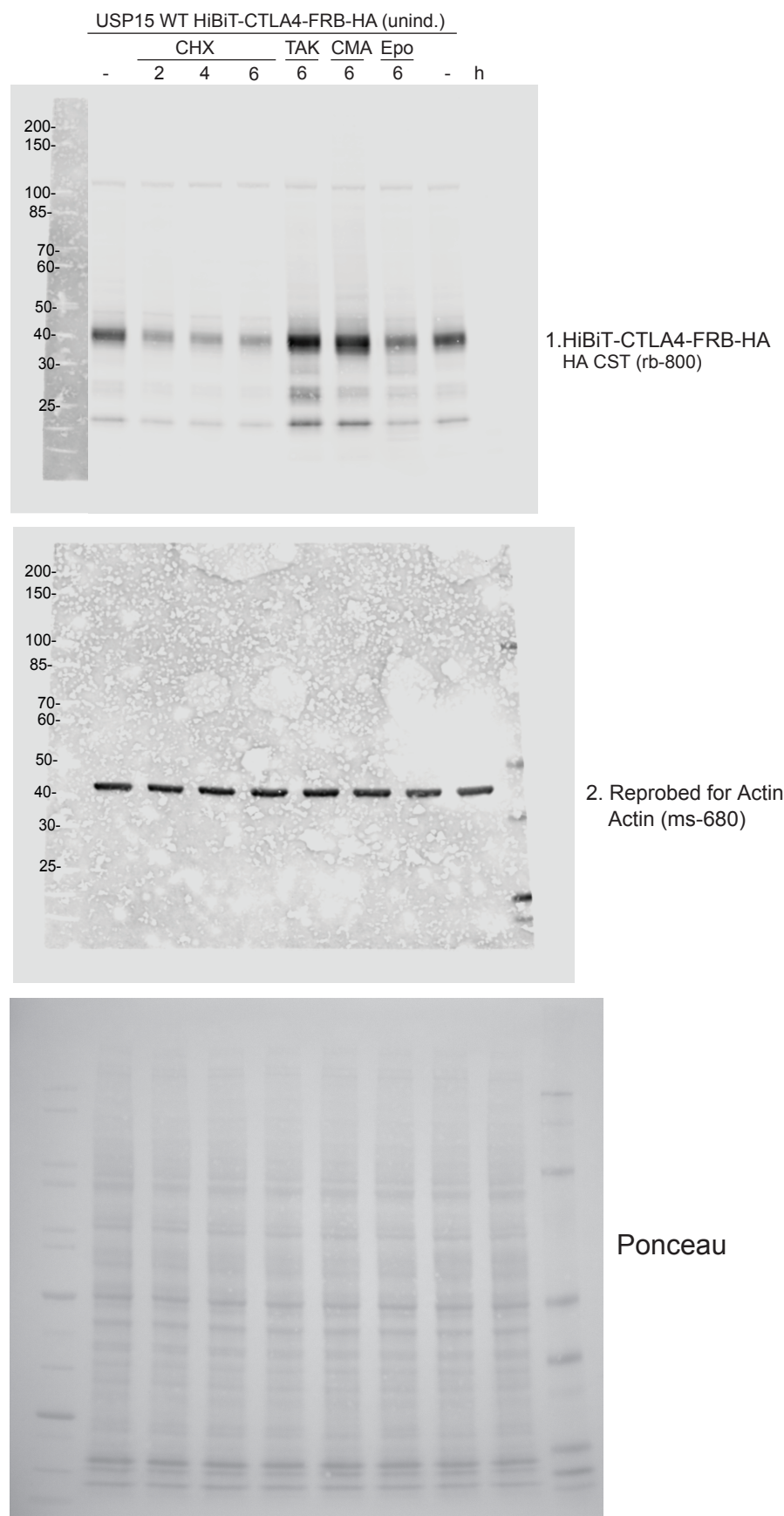

Figure S3D

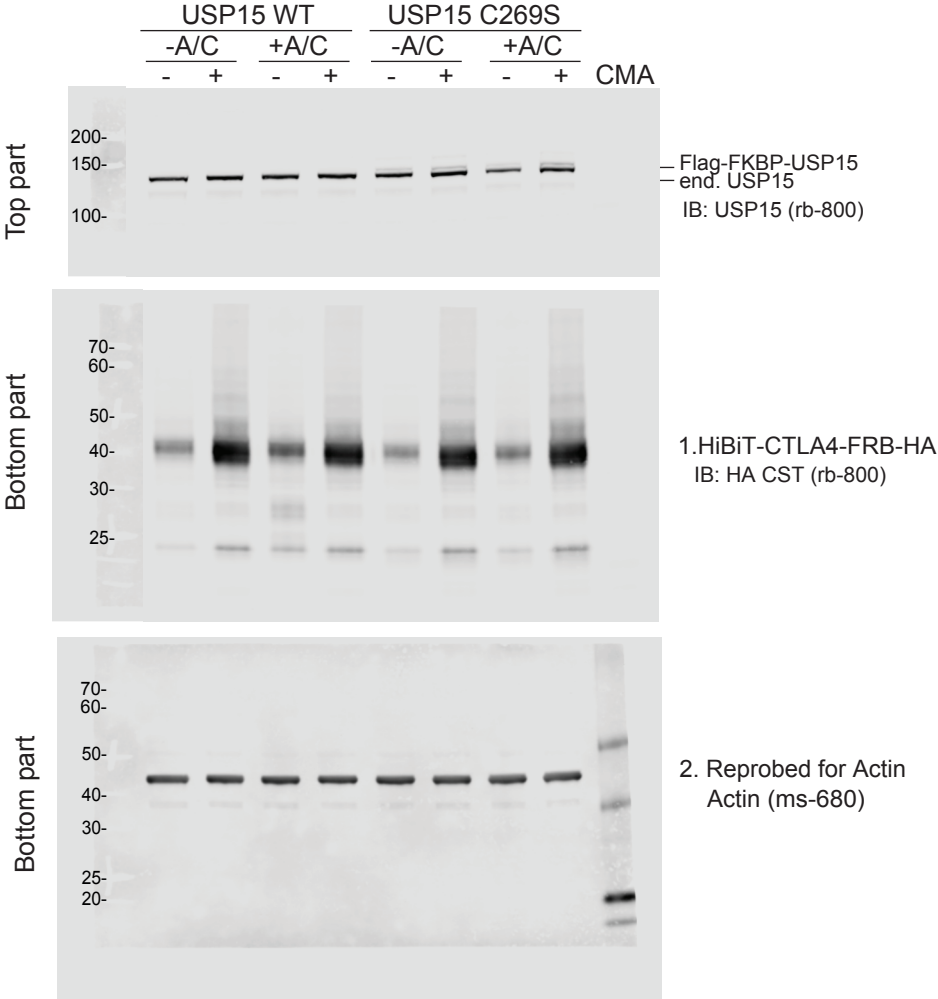

Supplement: Supplementary file 6 [file LSA-2025-03563_SdataFS3.pdf]

### Figure 6C

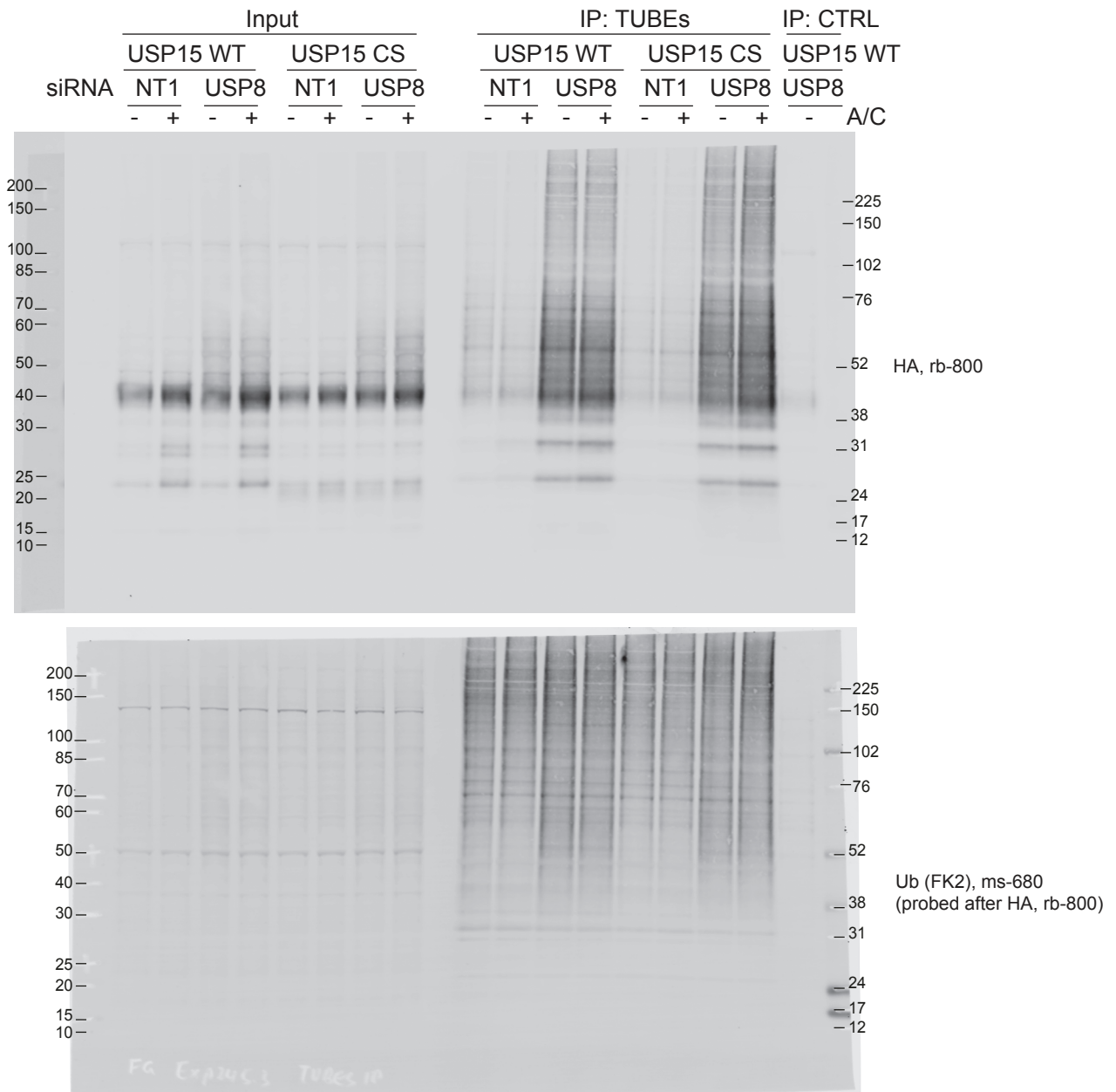

Figure 6C continued

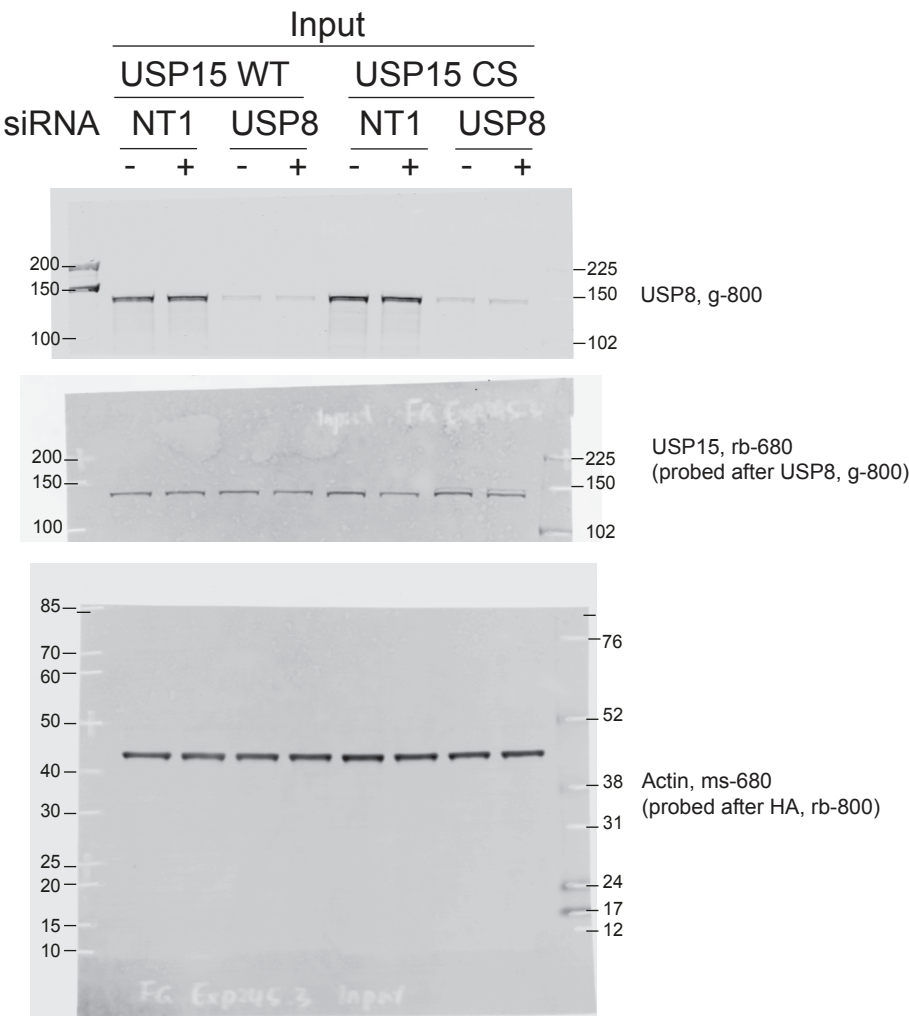

Supplement: Supplementary file 7 [file LSA-2025-03563_SdataF6.pdf]
